# Moderate consumption of freeze-dried blueberry powder increased net bone calcium retention compared with no treatment in healthy postmenopausal women: a randomized crossover trial

**DOI:** 10.1016/j.ajcnut.2023.05.033

**Published:** 2023-06-01

**Authors:** Joanna K. Hodges, Maria Maiz, Sisi Cao, Pamela J. Lachcik, Munro Peacock, George P. McCabe, Linda D. McCabe, Dennis P. Cladis, George S. Jackson, Mario G. Ferruzzi, Mary Ann Lila, Regan L. Bailey, Berdine R. Martin, Connie M. Weaver

**Affiliations:** 1 Department of Nutritional Sciences, the Pennsylvania State University, University Park, Pennsylvania, United States; 2 Department of Nutrition Science, Purdue University, West Lafayette, Indiana, United States; 3 School of Medicine, Indiana University, Indianapolis, Indiana, United States; 4 Department of Statistics, Purdue University, West Lafayette, Indiana, United States; 5 Department of Food Science and Technology, Virginia Polytechnic Institute and State University, Blacksburg, Virginia, United States; 6 Department of Physics and Astronomy, Purdue University, West Lafayette, Indiana, United States; 7 Arkansas Children's Nutrition Center, University of Arkansas for Medical Sciences, Little Rock, Arkansas, United States; 8 Department of Food, Bioprocessing and Nutrition Sciences, North Carolina State University, Kannapolis, North Carolina, United States; 9 Institute for Advancing Health through Agriculture, Texas A&M University, College Station, Texas, United States; 10 School of Exercise and Nutritional Sciences, San Diego State University, San Diego, California, United States

**Keywords:** bone, blueberries, calcium, osteoporosis, polyphenols, postmenopausal

## Abstract

**Background:**

Preclinical studies suggest that blueberry consumption is associated with improved bone health.

**Objectives:**

We conducted a blueberry dose-response study in ovariectomized (OVX)-rats that informed a study in postmenopausal women using the urinary appearance of calcium (Ca) tracers from prelabeled bone to reflect changes in bone balance. We hypothesized that blueberry consumption would reduce bone loss in a dose-dependent manner compared with no treatment.

**Methods:**

OVX rats were fed 4 doses of blueberry powder (2.5%, 5%, 10%, and 15%) in randomized order to determine bone ^45^Ca retention. Fourteen healthy, nonosteoporotic women ≥4 y past menopause were dosed with 50 nCi of ^41^Ca, a long-lived radioisotope, and equilibrated for 5 mo to allow ^41^Ca deposition in bone. Following a 6-wk baseline period, participants were assigned to a random sequence of 3 6-wk interventions, a low (17.5 g/d), medium (35 g/d), or high (70 g/d) dose of freeze-dried blueberry powder equivalent to 0.75, 1.5, or 3 cups of fresh blueberries incorporated into food and beverage products. Urinary ^41^Ca:Ca ratio was measured by accelerator mass spectrometry. Serum bone resorption biomarkers and urinary polyphenols were measured at the end of each control and intervention period. Data were analyzed using a linear mixed model and repeated measures analysis of variance.

**Results:**

In both OVX rats and postmenopausal women, blueberry interventions benefited net bone calcium balance at lower but not at higher doses. In women, net bone calcium retention increased by 6% with the low (95% CI: 2.50, 8.60; *P* < 0.01) and 4% with the medium (95% CI: 0.96, 7.90; *P* < 0.05) dose compared with no treatment. Urinary excretion of hippuric acid increased dose-dependently with blueberry consumption. No significant relationships were found between bone resorption biomarkers, 25-hydroxyvitamin D, and interventions.

**Conclusions:**

Moderate consumption (<1 cup/d) of blueberries may be an effective strategy to attenuate bone loss in healthy postmenopausal women.

This trial was registered at clinicaltrials.gov as NCT02630797.

## Introduction

Osteoporosis is the most common bone disease in older adults, characterized by low bone mass and increased fragility that can result in fractures at the spine, hip, or wrist [1]. Women at menopause undergo a major and rapid decrease in estrogen secretion, causing a loss in bone mass that adds substantially to the increased incidence of age-related osteoporotic fracture [2]. During this period, the hormonal imbalance results in an increase in oxidative stress, which increases bone resorption and decreases bone formation resulting in rapid bone loss [3,4]. Dietary flavonoid intakes were positively associated with bone mineral density (BMD) in a large sample of perimenopausal women [5,6].

Blueberries (Vaccinium spp.) are rich in polyphenols, including anthocyanins, phenolic acids, and flavan-3-ols [7]. These account for a total phenolic content ranging from 262 to 930 mg/100 mg fresh weight [8], depending on the cultivar. Most bone-related benefits of blueberries have been demonstrated in preclinical models of ovariectomy-induced (OVX) bone loss. In this model, blueberry (BB) powder at doses as low as 1%-10% of the diet (w/w) administered for 5-14 wk prevented bone loss as assessed by BMD, decreased bone resorption, increased bone formation, and improved bone histomorphometry [[9], [10], [11], [12]]. Several of the constituent polyphenols in blueberries were also shown to prevent bone loss in animal models. Oral administration of delphinidin, one of the major anthocyanidins in berries, at 10 mg/kg and 3 mg/kg significantly prevented bone loss in both the OVX- and the activator of NF-κB ligand (RANKL)-induced osteoporosis model [13].

More recent preclinical studies suggest that the metabolites arising from bacterial fermentation of BB polyphenolics may be, in part, responsible for the bone-protective effects of BB. For instance, 3-(3-hydroxyphenyl)-propionic acid (3-OH-PPA) in mice increased bone volume, trabecular thickness, and osteoblastic cell number while decreasing the number of osteoclasts in vivo, and promoted cell differentiation toward osteoblasts in vitro [14]. Hippuric acid derived from the serum of BB-fed rats decreased RANKL expression in bone marrow-derived stromal cells and decreased osteoclast resorptive activity [10]. Both 3-OH-PPA and hippuric acid (2 phenolic acid metabolites that appear in the serum of BB diet–fed rats in the highest concentrations [15]) from mice fed 10% BB diet suppressed osteoblast maturation, proliferation, and resorptive activity through RANKL-independent pathway [16], and increased bone mass dose-dependently [17].

The aims of our studies were as follows: *1)* to conduct a proof-of-principle study in rats to determine whether an approach of a randomized crossover design with an outcome measure of the urinary appearance of a calcium tracer from prelabeled bone would be successful in evaluating a dose-response effect of BB feeding on bone calcium balance and *2)* to use a similar approach in postmenopausal women to evaluate the dose-response effect of BB powder added to a regular diet on net bone calcium retention*.* We initially hypothesized a positive dose-response effect of blueberries on bone; however, the rat study suggested that lower doses would be more effective at reducing bone loss in postmenopausal women*.*

## Methods and Materials

### Preclinical study

#### Study design

This study was a randomized, crossover intervention to evaluate the dose-response effect of 2.5%, 5%, 10%, and 15% (w/w) freeze-dried BB diet on net bone calcium balance following the design previously reported [18] and shown in Supplementary Figure 1. Twenty 4-mo-old female OVX Sprague Dawley rats (Harlan Laboratories) were individually housed in stainless steel wire-bottom cages with a 12-h on-off cycle. Rats were allowed to acclimate to their new environment and stabilize from ovariectomy for 75 d. On d 45, they were dosed via a tail-vein injection with 63 microcuries (μCi) of ^45^Ca dissolved in 200 μl of sterile saline and allowed to equilibrate in bone. On d 65, 10 d prior to the determination of baseline ^45^Ca excretion, rats were changed from a chow diet to an AIN93-M polyphenol-free diet (soybean oil replaced with corn oil, Research Diets). The BB diets were modified from the polyphenol-free diet by adjusting the quantities of sugars, maltodextrin, and cellulose that the BB powder displaced. The freeze-dried BB powder was provided by the Wild Blueberry Association of North America and freeze-dried by FutureCeuticals, Inc. The diets were prepared and pelleted under low-temperature conditions to avoid polyphenol degradation (Research Diets, Inc). Upon arrival, the diet was stored at −80°C, and weighed out portions for each rat were transferred to −20°C during treatment periods.

Each intervention was provided for 10 d, followed by a 10-d nonintervention period. During baseline, intervention, and nonintervention periods, animals were placed in metabolic cages. Twenty-four h urine was collected, and ^45^Ca was measured with the use of Beckman LS 6500 Scintillation counter (Beckman Instruments Inc) and total calcium by atomic absorption spectrometry (AAnalyst 300, PerkinElmer Instruments) to determine the urinary ^45^Ca:Ca ratio.

### Statistical analyses

To determine changes in bone balance (net calcium retention), the ratio of urinary ^45^Ca to total Ca was transformed using the natural logarithm to correct for skewness. Differences between the predicted values from a regression line created from nonintervention data points and observed values during the intervention periods were used to calculate percent change and 95% CIs. The method has been validated and described previously [19]. To determine differences among treatments on rat weight, weight gain, food intake, and feeding efficiency, a one-factor ANOVA was performed using JMP 12 (SAS Institute).

### Human study

#### Participants

**FIGURE 1:**
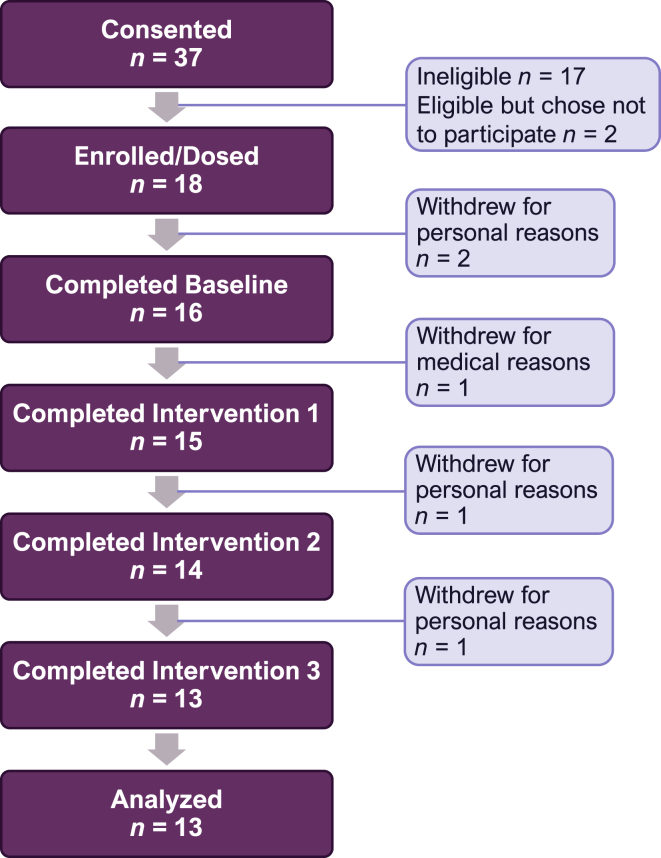
CONSORT chart. The chart shows participant progress through the study phases.

Thirty-five postmenopausal women aged 45–70 y who were >4 y post natural menopause or had a total hysterectomy were recruited from the Lafayette, IN area with the use of fliers and online advertisements between February and June 2017. Each woman signed a written consent form and completed a screening that included height and weight measurements, a brief medical history, diet, and physical activity assessment. All participants were healthy, as verified by serum biochemistries, including a comprehensive metabolic panel. Exclusion criteria included having a history of metabolic bone disease, personal history or family history of low trauma fractures, personal history of cancer, thromboembolisms, clotting disorders, uncontrolled hypertension, uncontrolled thyroid function, malabsorption syndrome, seizure disorders, heart attack, or severe obesity (BMI > 35 kg/m^2^). Subjects taking any medications for osteoporosis, including HRT or glucocorticoids within 6 mo or bisphosphonates within 2 y of the study and those who declined to discontinue the use of supplements of their own selection were also excluded. Seventeen women were shown to be ineligible for medical reasons, and 2 women decided not to participate because of personal reasons (Figure 1).

Eighteen women received an intravenous injection of 50 nCi of ^41^Ca dissolved in bacteriostatic saline (Federal Institute of Technology, ETH Zurich) between May and June 2017. The dose was provided by nursing staff at Indiana University Health University Hospital. All doses were tested for sterility and pyrogenicity prior to administration. All other clinical visits occurred in the Clinical Research Center at Purdue University. Four mo after dosing, participants were instructed to discontinue any vitamin and mineral supplementation of their own selection and take a daily multivitamin provided by the study (Spectravite Advanced Formula; CVS/Pharmacy), which contained 200 mg Ca/d and 800 IU vitamin D/d to protect against very low-calcium intakes that may confound study outcomes. This study was approved by the Institutional Review Board of Purdue University (approval no. 1405014899) and the Institutional Review Board of Indiana University (approval no. 1607510714) and was registered at clinicaltrials.gov as NCT02630797.

### Intervention products

Freeze-dried BB powder was a composite of several wild genotypes of intact fresh berries harvested in the Northeast United States and Canada (WBANA blueberry powder WBA1.1; FutureCeuticals). The BB powder was incorporated into food matrices, including granola bars, spread, and drinks consumed daily as part of a self-selected diet. The product composition, energy, macronutrient, and polyphenol contents are shown in Table 1. The methods of ensuring the stability of polyphenols and product palatability were described previously [20].

**TABLE 1:** Ingredients, nutrients, and polyphenol profiles of the intervention products (granola bites, spread, and lemonade) containing freeze-dried BB powder

	Granola (per low dose serving – 90 g)	Spread (per low dose serving – 90 g)	Lemonade (per low dose serving – 318 g)
Ingredients			
	BB powder (17.5 g)	BB powder (17.5 g)	BB powder (17.5 g)
	White sugar and water syrup	Whipped cream cheese spread	Lemonade, low calorie, with aspartame, powder dissolved in water
	Granola (oats n’ honey protein by Nature Valley)	Water, noncarbonated	
	Regular creamy peanut butter by Jif		
	Water, noncarbonated		
Energy (kcal)	281	171	68
Carbohydrate (mg)	11	20	1
Protein (mg)	8	3	0.3
Fat (mg)	46	11	17
Fiber (mg)	5	2.5	2.5
Calcium (mg)	42	51	90
Magnesium (mg)	0.1	0	3
Phosphorus (mg)	0	0	39
Potassium (mg)	200	81	84
Sodium (mg)	146	205	16
Anthocyanins (mg)	95 ± 11	97 ± 6	95 ± 11
Phenolic acids (mg)	126 ± 19	116 ± 16	118 ± 5
Flavan-3-ols (mg)	2.5 ± 0.1	2.8 ± 0.3	2.3 ± 0.3
Flavonols (mg)	83 ± 5	81 ± 5	88 ± 2
Total phenolics (mg)	607± 145	522 ± 36	642 ± 34

Abbreviation: BB, blueberry.

### Study design

**FIGURE 2:**
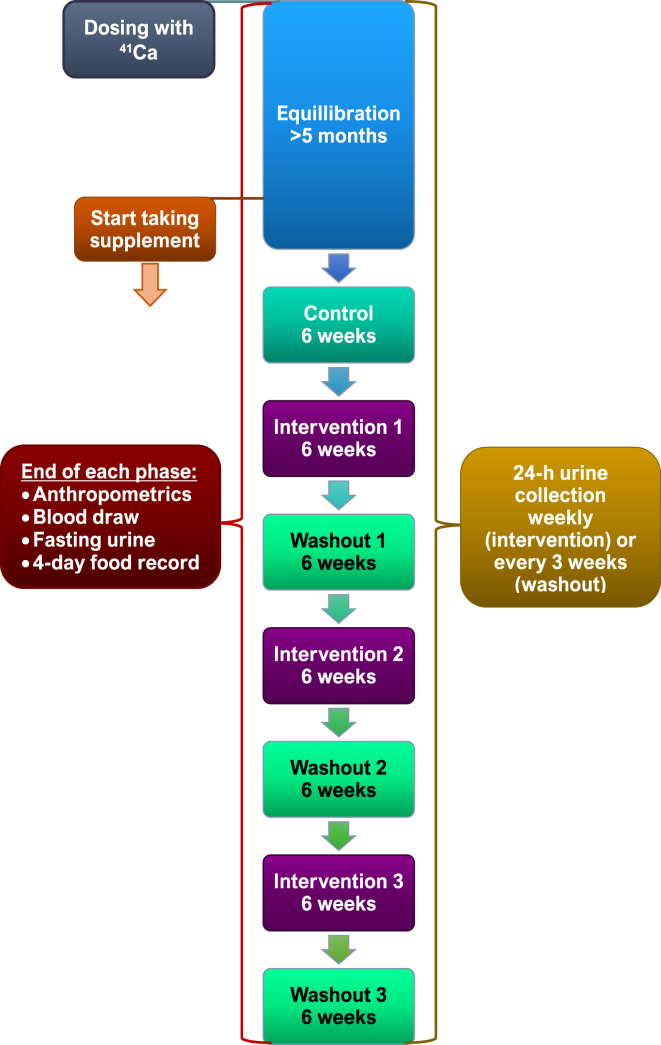
Human study design. Participants were dosed with ^41^Ca and completed a 5-mo equilibration, a 6-wk baseline, and a random sequence of 3 interventions, each followed by a 6-wk washout. Urine was collected over 24 h weekly during the intervention and every 3 wk during washout for the assessment of primary outcome, net bone calcium retention. Secondary outcomes, including bone metabolism biomarkers, serum calcium, vitamin D, and urinary polyphenolics, were measured in fasting blood collected at the end of each study period.

This study was a double-blind, crossover, randomized trial with a 6-wk baseline period and 3 intervention periods followed by washout periods, each lasting for 6 wk (Figure 2). After enrollment, participants traveled to Indiana University, where they received a 50-nCi intravenously administered dose of ^41^Ca [21]. One participant who was dosed with ^41^Ca 4 y before the start of the study had enough for detection; therefore, a second dose was omitted. The isotope was allowed to equilibrate with whole-body calcium for 5 mo. During equilibration, participants collected 24-h urine monthly and biweekly during the subsequent baseline period to monitor ^41^Ca urinary excretion.

After baseline, participants were randomly assigned to a sequence of 3 intervention periods, each corresponding to a low (17.5 g/d), medium (35 g/d), or high (70 g/d) dose of freeze-dried BB powder equivalent to 0.75, 1.5, or 3 cups of fresh blueberries. Each intervention lasted for 6 wk and was followed by a 6-wk washout period, determined to be optimal by simulation modeling of data from prior studies. Participants were instructed to maintain their usual exercise and dietary habits during the study. The intervention products were provided in a double-blind manner between February and November 2018. Products were coded by research staff prior to dispensing them to the participants by the study coordinator. Unconsumed foods were collected weekly during each intervention to monitor adherence. Participants received a list of polyphenol-rich foods to avoid and/or limit. Participants were instructed to complete a 4-d dietary record at the end of baseline and each intervention and washout period to quantify habitual intakes of energy and bone-relevant nutrients. The records were analyzed with the use of the Nutrition Data System for Research (2018 ed.; University of Minnesota). Participants collected 24-h urine weekly during the intervention and every 3 wks during washout to monitor ^41^Ca excretion. Urinary creatinine concentrations were measured in 24-h urine to check for collection completeness. To minimize attrition and maintain adherence, participants received weekly e-mail reminders and calendars to check off all completed specimen collections, questionnaires, and dietary records.

During the trial, one participant reported that the BB drink irritated a mouth sore, which was resolved by diluting the drink. The institutional review board suspended the study to amend the consent form to include a statement that consumption of products with BB powder may cause oral irritation. The phases interrupted by suspension were repeated (*n* = 3). Specimen collection for this study ended in January 2019, and there was no further interaction with the participants.

### Bone density and anthropometrics

At baseline, the BMC and BMD were measured at the hip, spine, and total body with the use of DXA (Lunar iDXA device, GE Medical Instruments) to characterize the cohort prior to the intervention. At the terminal clinical visit of each study period (baseline, intervention, and washout), waist circumference, standing and sitting height were measured with the use of a wall-mounted stadiometer, and fasting weight with the use of a digital scale.

### Net bone calcium retention

Net bone calcium retention, our primary outcome, was assessed with the use of the urinary ^41^Ca: total Ca ratio. The validity of this method, as applied to monitoring changes in BMD, was described in detail by Weaver et al. [19]. Urinary ^41^Ca was measured with the use of accelerator MS at Purdue Rare Isotope Measurement Laboratory, as previously reported [22].

### Serum calcium, vitamin D, and biochemical markers of bone metabolism

Prior to each intervention, serum calcium was measured with the use of atomic absorption spectrometry (AAnalyst 300, PerkinElmer Instruments), and serum 25(OH)D was measured with the use of HPLC. At baseline and the end of each intervention period, fasting blood and urine were collected in the morning to assess serum calcium, 25(OH)D, and biomarkers of bone metabolism: serum IGF-1, IGF-binding protein-3 (IGFBP-3), and their ratio, procollagen type 1 N-terminal propeptide (P1NP), cross-linked C-telopeptide of type II collagen (CTx-II), sclerostin, osteoprotegerin (OPG), receptor activator of NF-κB ligand (RANKL), and their ratio, as well as urinary N-terminal telopeptide (NTx), normalized to creatinine. PTH was also assessed at baseline. Bone metabolism biomarkers and PTH were assessed with the use of commercial ELISA kits. Urinary creatinine was measured with the use of an enzymatic colorimetric assay (Roche Diagnostics Ltd.).

### Urinary polyphenolics

Polyphenolics and their metabolites, including total phenolics, flavanols, phenolic acids, and anthocyanin metabolites, were assessed, as previously described [23], in 24-h urine collected at the end of baseline and each intervention period and normalized to creatinine to assess participant adherence to study protocol.

### Statistical analyses

A sample size of 18 was recruited to retain 13 and to give 80% power to detect a minimum difference of 9% in net bone calcium retention. This effect size was based on our previous studies using nutritional interventions with a primary outcome of bone calcium retention [19,21]. The primary response variable in this study was the log of the ^41^Ca:Ca ratio. For each subject, a simple linear regression model was fit through all nontreatment values to create a regression line, and the predicted ^41^Ca:Ca ratio for each 24-h urine collection during the intervention was subtracted from the observed value. The means of these differences were computed for each individual and averaged across individuals to provide estimates of treatment effects. Results were back transformed to the original scale and expressed in terms of net bone calcium retention. Bootstrap resampling was used to calculate 95% CIs. Our bootstrap had 2 levels of sampling, i.e., sampling subjects (to assess variation between subjects) and sampling residuals (to assess within-subject variation). After obtaining the prediction curves for each subject and collecting all the residuals from the model used to obtain these curves, we first performed with-replacement sampling from the subjects 14 times. Then, we applied random selection with the replacement of residuals from the original model to obtain a bootstrap data set. We performed this procedure 1000 times, and from each bootstrap data set, we obtained an estimate for % bone calcium retention. We then used these estimates to obtain bootstrap CIs from the empirical bootstrap distributions for each treatment. To examine the effect of BB dose, the 3 treatments were analyzed in a repeated measures mixed model that included treatment, serum polyphenol concentrations, and the interaction. Linear mixed models were used to test participant characteristics, serum polyphenols, and biochemical bone markers to predict response in % bone calcium retention. Bone biochemistry data (RANKL, RANKL/OPG ratio, and P1NP) and urinary polyphenolics and their metabolites were analyzed by RM ANOVA followed by Dunnett’s post hoc test. Data were analyzed with the use of SAS software (version 9.1 SAS Institute). Statistical significance was set at *P* value of <0.05.

## Results

### Preclinical study

**FIGURE 3:**
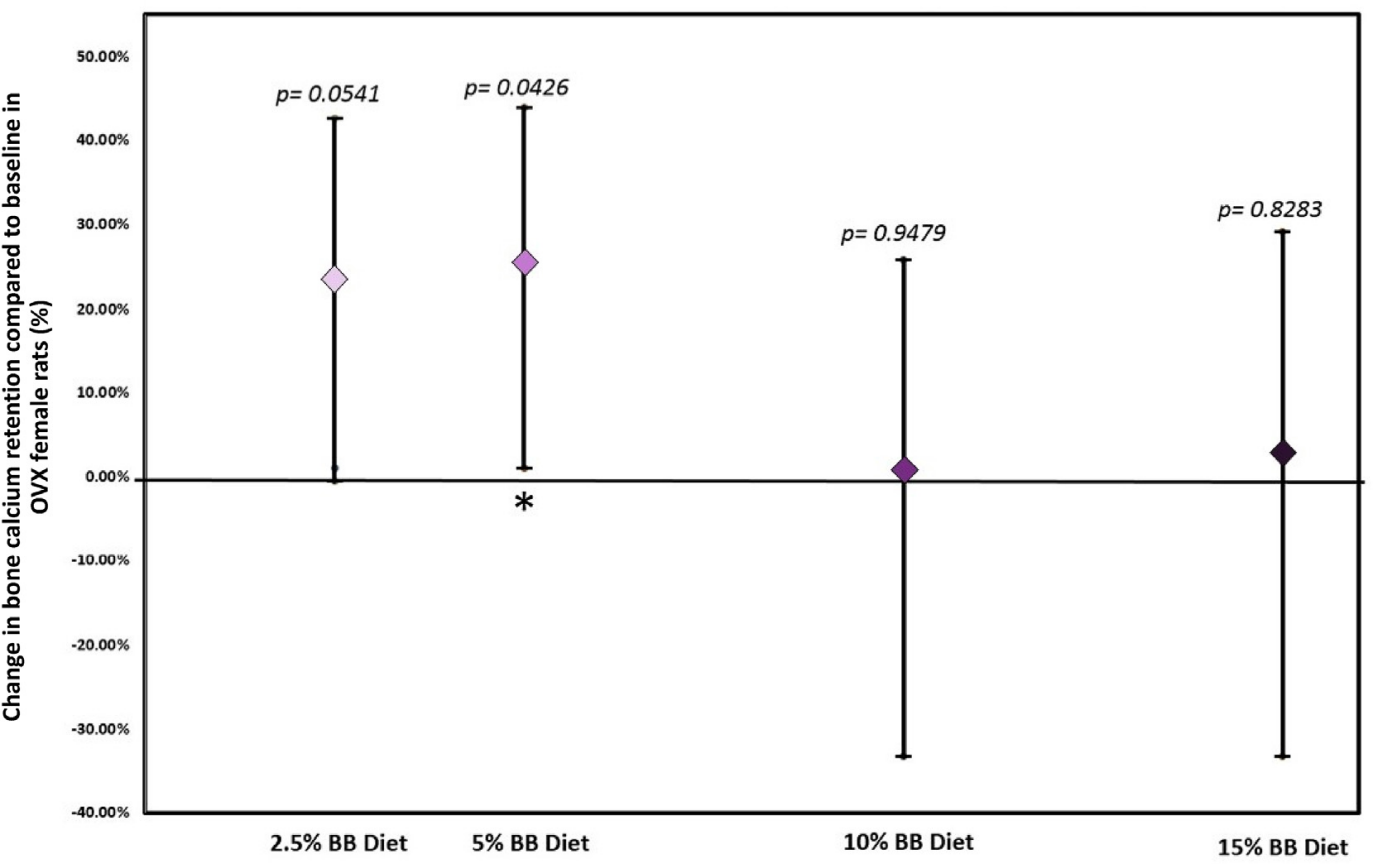
Hormetic effect of BB powder on bone balance in 20 OVX female rats. The net bone calcium retention increased with the lowest dose of BB power, showed a trend toward an increase with the moderate dose, and no effect with the highest dose compared with no treatment. Data are means and 95% CI, ∗*P* < 0.05, *n* = 20. BB, blueberry; OVX, ovariectomized.

The proof-of-principle study in rats was successful. No significant differences were observed in body weight, weight gain, food intake, or food efficiency ratio due to BB feeding. Bone balance or net calcium retention increased by 25.6% (*P* = 0.0426) on the 5%, by 24% on the 2.5% (*P* = 0.0541) and failed to increase on the higher doses of 10% and 15% BB (w/w) diets (Figure 3).

### Human Trial

#### Participant characteristics at baseline

Thirteen healthy women completed the entire study, and one woman completed 2 out of 3 interventions (Figure 1). Data from 13 participants (*n* = 13) were used for the analysis of the primary outcome: net bone calcium retention. Participants were, on average, 7 y past menopause and had BMI slightly above the normal range (Table 2). Three of the women had undergone hysterectomy, and all women were past the stage of rapid menopausal bone loss. Participant BMD was comparable with that of the US female non-Hispanic White population aged 50–79 y [24,25]. The PTH concentration in blood was within the normal range (10–65 pg/mL) for all participants. Based on the 4-d dietary records, participants' mean baseline intakes of calcium, vitamin D, magnesium, sodium, and potassium were comparable to mean intakes within the United States and below (above for sodium) the dietary recommendations for these nutrients [26].

**TABLE 2:** Participant characteristics at baseline

Characteristic	Mean ± SEM
Age (y)	56 ± 5
Race (self-reported using NIH classifications)	
White	*n* = 12
Black	*n* = 1
Asian	*n* = 1
BMI (kg/m^2^)	26 ± 4
Waist circumference (cm)	98 ± 2
Time past menopause (y)	6.7 ± 3.6
BMD, Total body (g/cm^2^)	1.13 ± 0.03
BMC, Total body (g)	2208 ± 320
BMD, Femoral neck (g/cm^2^)	0.89 ± 0.03
T-score, Femoral neck	−1.11 ± 0.24
BMD, Lumbar spine (g/cm^2^)	1.10 ± 0.04
T-score, Lumbar spine	−0.71 ± 0.32
FM, %	40 ± 3
PTH (pg/mL)	47 ± 16
Dietary intake of:	
Calcium (mg)	969 ± 67
Vitamin D (calciferol, IU)	320 ± 80
Magnesium (mg)	288 ± 23
Sodium (mg)	2665 ± 256
Potassium (mg)	2267 ± 166

Data are means ± SEM, *n* = 14. Concurrently with self-selected diets, participants were instructed to consume a daily multivitamin containing 200 mg of calcium, 800 IU of vitamin D, and 50 mg of magnesium. Abbreviations: BMC, bone mineral content; BMD, bone mineral density; BMI, body mass index; PTH, parathyroid hormone.

#### Adherence to intervention and intake of bone-relevant nutrients

The amount of unconsumed intervention products brought back to the clinic was very low. The participants consumed 98% of the foods and 92% of the multivitamin supplements. During the study, participant intakes of energy, carbohydrate, soluble, and insoluble fiber were significantly higher during the intervention with a high dose (70 g/d) of BB powder compared with baseline (Supplementary Figure 2). Intakes of vitamin D (including the amount provided in the study supplement) were lower during the treatment compared with baseline, regardless of the BB powder dose. Participants were weight-stable throughout the study. No significant changes were found in fasting weight, BMI, waist circumference, or standing and sitting height due to treatment or dose of BB powder.

#### Net bone calcium retention

**FIGURE 4:**
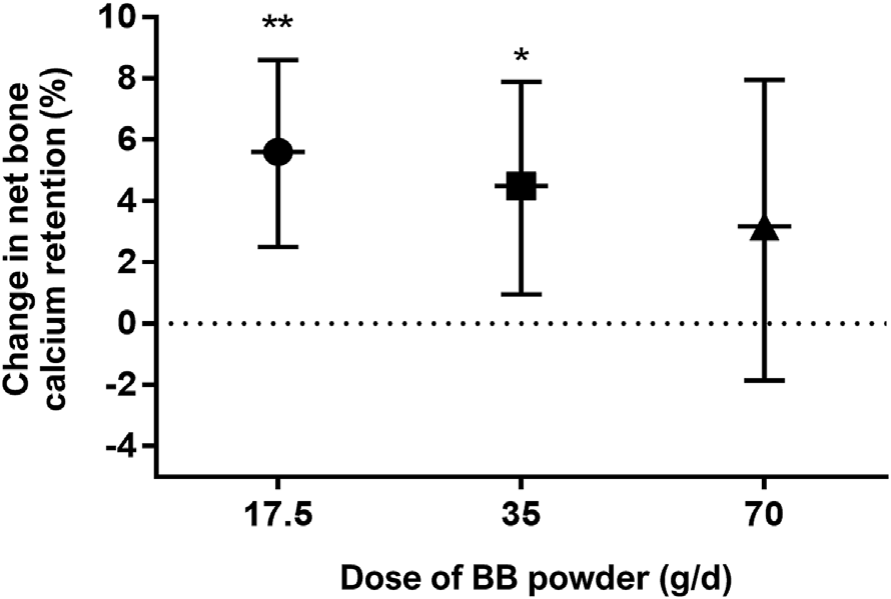
Change in net bone calcium retention as measured by urinary ^41^Ca:Ca ratio in postmenopausal women consuming low (17.5 g/d), medium (35 g/d), and high dose (70 g/d) of freeze-dried BB powder compared with no treatment. Net bone calcium retention is expected to increase above the control line (dotted line) whenever a treatment reduces bone resorption. Our results showed that the net bone calcium retention increased with the low and medium doses of BB power compared with no treatment. Data were analyzed with the use of a linear mixed model. Data are means and 95% CI, ∗*P* < 0.05, ∗∗*P* < 0.01, *n* = 13. BB, blueberry.

Participants consuming the low (17.5 g/d) and medium (35 g/d) doses of BB for 6 wk retained significantly more calcium in bone compared with no treatment (Figure 4). The increase in bone calcium retention over the nonintervention periods was 5.6% (*P* < 0.01) and 4.5% (*P* < 0.05) for the low and medium doses, respectively. There was no significant relationship between the high dose (70 g/d) of BB powder and net bone calcium retention (*P* = 0.19). In participants consuming the high dose for 6 wk, net bone calcium retention was 3.2% over the nonintervention periods. None of the variables included in the linear mixed models (participant characteristics, serum polyphenols, and bone biomarkers) predicted the response of % bone calcium retention.

#### Serum calcium, vitamin D, and biochemical markers of bone metabolism

**FIGURE 5:**
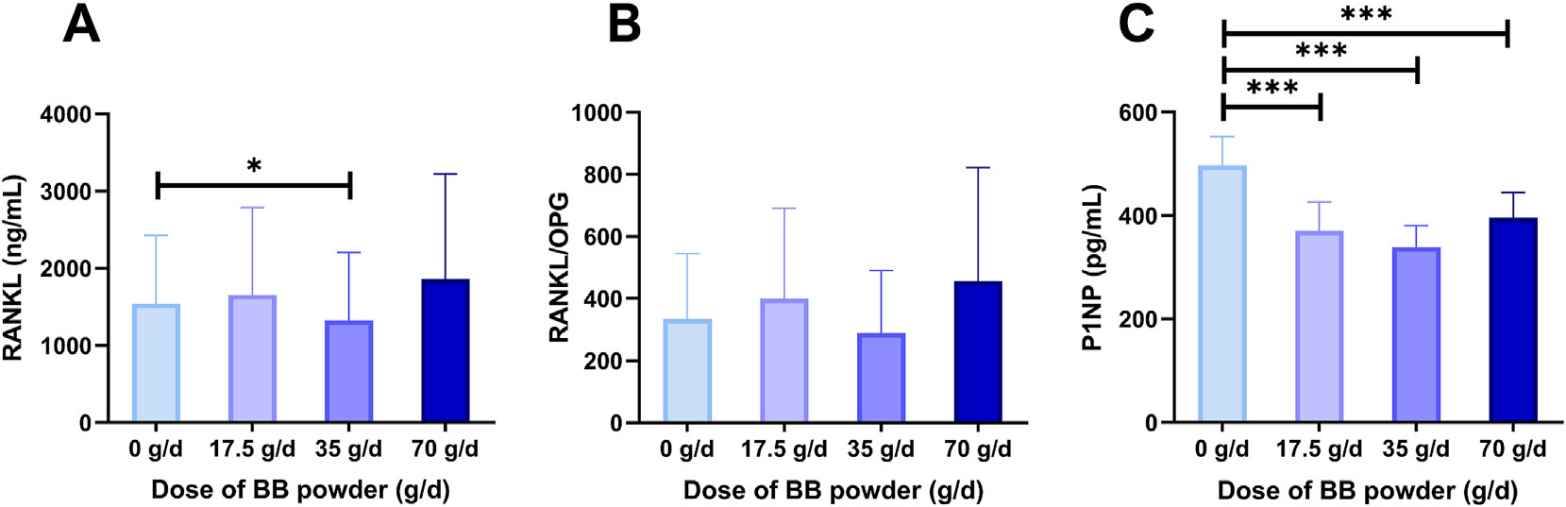
Serum RANKL(A), RANKL/OPG ratio (B), and P1NP concentration in postmenopausal women at baseline (0 g/d) and after the intervention with low (17.5 g/d), medium (35 g/d), and high dose (70 g/d) of freeze-dried BB powder. Bone resorption marker RANKL was significantly reduced by the medium dose of BB powder. Bone formation marker P1NP was reduced by the low, medium, and high doses of BB powder. Data were analyzed by RM ANOVA followed by Dunnett’s post hoc test. Data are means ± SD, ∗*P* < 0.05, ∗∗*P* < 0.01, *n* = 13. BB, blueberry; OPG, osteoprotegerin; P1NP, procollagen type 1 N-terminal propeptide; RANKL, receptor activator of nuclear factor kappa-Β ligand.

Serum calcium and 25(OH)D concentrations remained constant throughout the study regardless of treatment. The medium dose of BB powder reduced serum concentrations of bone resorption agonist RANKL by 14% (*P* < 0.05) but not the RANKL/OPG ratio (Figure 5). The low, medium, and high doses of BB powder also decreased concentrations of serum P1NP, a marker of bone formation, by a mean of 26% (*P* < 0.01). No significant relationships were found between the treatment and markers of bone resorption: serum IGF-1, IGFBP-3, and their ratio, sclerostin, OPG, CTx-II, and urinary NTx normalized to creatinine (Table 3). The mean creatinine concentration (54.0 ± 24.9 mg/dL) varied both within and between participants ranging from 18.60 mg/dL to 94.80 mg/dL. There was no correlation between urinary creatinine, treatment, or dose of BB powder.

**TABLE 3:** Fasting serum and urinary bone metabolism biomarkers, serum calcium, and serum vitamin D (25(OH)D) by intervention in postmenopausal women at baseline and after low, medium, and high doses of freeze-dried BB powder.

	Baseline	Low	Medium	High
	(0 g/d)	(17.5 g/d)	(35 g/d)	(70 g/d)
Calcium (mg/dL)^1^	37.2 ± 6.9	10.03 ± 0.54	10.24 ± 0.59	10.18 ± 0.45
25(OH)D (ng/mL)^1^	10.10 ± 0.46	36.8 ± 6.5	36.4 ± 5.9	36.0 ± 5.5
**Hormones**				
IGF-1 (ng/mL)	106.6 ± 32.7	100.6 ± 29.1	100.8 ± 33.5	104.6 ± 36.5
IGFBP-3 (μg/mL)	2.06 ± 0.26	2.10 ± 0.36	2.08 ± 0.36	2.11 ± 0.33
IGF-1/IGFBP-3 (ng/μg/mL)	51.8 ± 14.6	48.0 ± 11.7	48.5 ± 13.2	49.1 ± 12.6
**Bone resorption markers**				
CTx-II (pg/mL)	454.3 ± 141.9	597.4 ± 405.7	540.4 ± 306.6	671.0 ± 496.3
NTx (nM BCE/mM Cr)	141.1 ± 107.7	162.7 ± 119.5	134.2 ± 121.5	165.8 ± 120.3
**Osteocyte activity markers**				
OPG (pmol/L)	5.02 ± 1.02	4.75 ± 0.97	5.08 ± 1.18	5.26 ± 1.25
Sclerostin (ng/mL)	0.79 ± 0.22	0.83 ± 0.21	0.85 ± 0.20	0.86 ± 0.18

Data were analyzed by RM ANOVA followed by Dunnett’s post hoc test. Data are means ± SD, *n* = 13. No statistical significance was observed in any of the tabulated outcomes.

Abbreviations: BB, blueberry; BCE, bone collagen equivalents; CTx-II, C-telopeptide of type II collagen; IGF-1, insulin-like growth factor 1; IGFBP-3, insulin-like growth factor-binding protein 3; NTx, N-terminal telopeptide; OPG, osteoprotegerin.

1 Serum calcium and 25(OH)D were assessed at baseline and prior to each intervention phase.

#### Urinary polyphenolics

**FIGURE 6:**
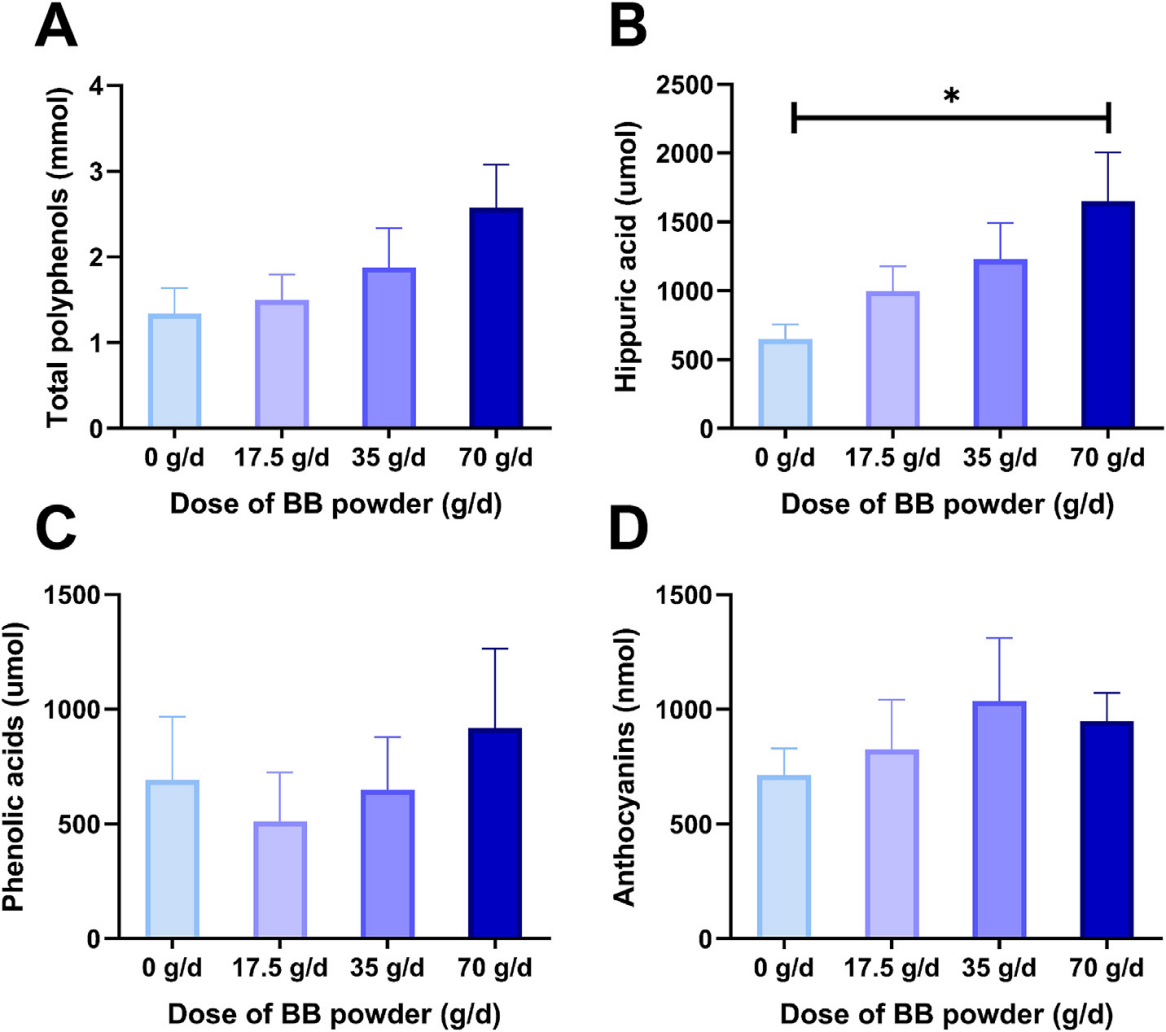
Urinary polyphenolics and their metabolites: total polyphenols (A), hippuric acid (B), phenolic acids (C), and anthocyanins (D) normalized to creatinine in postmenopausal women without (0 g/d) and after the intervention with low (17.5 g/d), medium (35 g/d), and high dose (70 g/d) of freeze-dried BB powder. Urinary excretion of hippuric acid was higher during the high-dose intervention compared with baseline. Data were analyzed by RM ANOVA followed by Dunnett’s post hoc test. Data are means ± SD, ∗*P* < 0.05, ∗∗*P* < 0.01, *n* = 14. BB, blueberry.

Based on the concentration of polyphenols and their metabolites measured in 24-h urine, the urinary excretion of hippuric acid was higher with the high-dose intervention compared with baseline (Figure 6). There was no correlation between the urinary excretion of other polyphenols or their metabolites, although a tendency toward a dose-dependent pattern of excretion was noticeable for total polyphenols.

## Discussion

Freeze-dried BB powder at lower to moderate doses, but not higher doses, increased bone calcium balance in both OVX rats and healthy postmenopausal women. The rat study informed the doses chosen in the clinical study. In postmenopausal women, the effective doses were 17.5 g/d (best) and 35 g/d. The low dose BB improved bone balance by 6%, the equivalent of 41 g of total body calcium. In addition, a bone resorption marker RANKL was significantly reduced by the medium dose of BB powder. Bone formation marker P1NP was also reduced, suggesting a reduction in bone turnover overall, although it did not predict the urinary ^41^Ca excretion, likely due to the higher variance in bone biomarkers as indicators of bone turnover compared with the more sensitive ^41^Ca:Ca ratio. Neither the consumption of BB powder nor its dose influenced other bone metabolism markers, serum calcium, or 25(OH)D. The consumption of BB powder dose-dependently increased the urinary excretion of hippuric acid, a common end-stage metabolite of several BB polyphenols, and a stimulator of osteoblast maturation and proliferation [16].

The lack of linear dose-response relationship is consistent with a report in OVX rats that a 100-d treatment with 5% (w/w semipurified diet) powdered freeze-dried blackberry, another polyphenol-rich fruit with similar phenolic content as BB, resulted in modest protection of tibial and femoral BMD, but no effect was seen with the higher dose of 10% [4]. This is indicative of a hormetic effect [27], whereby low doses provide a beneficial effect, whereas higher doses exert either a null or negative effect.

There are several possible mechanisms by which the hormetic effect could be mediated, including cellular redox status, bone osteoblast, and osteoclast activities, and changes in gut microbial composition. The high levels of polyphenols in BB are thought to be the bioactive components that might act as antioxidants, reducing oxidative stress and, therefore, preventing bone loss. Sato et al. [12] showed that BB increased the endogenous antioxidant response by bypassing the traditional antioxidant transcription factor Nrf2 in female but not male mice, and the effect was BB cultivar dependent. BB feeding also protected from estrogen deficiency, preserved bone, skeletal muscle, and body composition, and elicited antioxidant defense responses independently of classical antioxidant/estrogenic signaling. Similarly, BB increased both Alpha (within sample) and Beta (among sample) diversity of the gut microbiome and increased the prevalence of the taxon Ruminococcus1 in females but not males. Cladis et al. [23] evaluated the dose-response effect of BB polyphenols on gut microbial and phenolic metabolite profiles in OVX rats. Both Alpha and Beta diversity of gut microbial populations increased with low to moderate doses of BB polyphenols but decreased with high doses, indicating a hormetic effect of BB polyphenols on the gut microbiota. Changes in microbial diversity and phenol metabolites were highly correlated, suggesting a potential interaction between host and gut microbiome metabolism, which may be disrupted at high doses of BB phenols. The authors speculated that higher doses of BB polyphenols might saturate metabolite pathways in the lower gut. In a subsequent study, freeze-dried BB at a dose equivalent to 1-2 cups of fresh BB in humans did not benefit BMD or bone mechanical properties [28], consistent with the finding that higher doses do not offer additional benefits. Disruption of microbial metabolism at high doses may provide a clue to the mechanism underlying the hormetic effect observed in the present study, whereby high doses of BB have a negative effect on the gut microbiota and, subsequently, a null effect on bone health. Future studies to investigate the mechanisms causing the hormetic response are needed to evaluate this hypothesis.

### Strengths and limitations

Strengths of this paper include an agreement in the hormesis effect of BB with an animal model of postmenopausal women and a clinical study of postmenopausal women, the continued monitoring of a highly sensitive measure of bone balance, and mechanistic measures of polyphenol metabolites and gut microbiome profiles. The crossover design minimizes the potential confounding of the effect of BB on bone. Limitations include the use of a small convenience sample that lacked racial diversity and representation of the general population, thus, limiting the generalizability of the results. The long-term effects of BB consumption at different doses remain to be elucidated among postmenopausal women.

In conclusion, daily consumption of foods containing freeze-dried BB powder in the amount equivalent to 0.75 and 1.5 cups of blueberries, but not 3 cups, increased net bone calcium retention, indicative of a hormetic response and/or a possible negative gut microbiome-polyphenol interaction at higher doses. The effectiveness of a serving of blueberries in attenuating bone loss in healthy postmenopausal women demonstrates that a relatively short and accessible dietary intervention may prevent or delay bone loss without the side effects of conventional pharmacologic therapies. This practical dietary approach with food that already has a health connotation for consumers is likely to be well received by the public seeking osteoporosis prevention strategies.

## Funding

The research leading to these results was supported by grants to CMW from NIH/NCCIH (5R01AT008754) and RLB from Project Development Team within the ICTSI NIH/NCRR (UL1TR001108)

## Conflict of interest

The authors declare no conflict of interest.

## Data Availability

The data described in the manuscript, code book, and analytic code will be made available upon request, pending approval by the authors.

## Appendix A Supplementary data

Multimedia component1 Multimedia component1
