# Supplementary material for: Moderate consumption of freeze-dried blueberry powder increased net bone calcium retention compared with no treatment in healthy postmenopausal women: a randomized crossover trial
Source: Am J Clin Nutr. 2023 Jun 1;118(2):382–90. doi: 10.1016/j.ajcnut.2023.05.033 (PMC10447493; doi:10.1016/j.ajcnut.2023.05.033)
Supplement: Multimedia component1 [file mmc1.docx]

Moderate Consumption of Freeze-dried Blueberry Powder Increased Net Bone Calcium Retention in Healthy Postmenopausal Women: A Randomized Crossover Trial

Joanna K. Hodges^a^, Maria Maiz^b^, Sisi Cao^j^, Pamela J. Lachcik^b^, Munro Peacock^c^, George P. McCabe^d^, Linda D. McCabe^b^, Dennis P. Cladis^e^, George S. Jackson^f^, Mario G. Ferruzzi^g^, Mary Ann Lila^h^, Regan L. Bailey^i^, Berdine R. Martin^b^, and Connie M. Weaver^j,$^

^a^Department of Nutritional Sciences, The Pennsylvania State University, University Park, PA 16802

^b^Department of Nutrition Science, Purdue University, West Lafayette, IN 47907

^c^School of Medicine, Indiana University, Indianapolis, IN 46202

^d^ Department of Statistics, Purdue University, West Lafayette, IN 47907

^e^Department of Food Science and Technology, Virginia Polytechnic Institute and State University, Blacksburg, VA 24061

^f^Department of Physics and Astronomy, Purdue University, West Lafayette, IN 47907

^g^Arkansas Children's Nutrition Center, University of Arkansas for Medical Sciences, Little Rock, AR 72202

^h^Department of Food, Bioprocessing and Nutrition Sciences, North Carolina State University, Kannapolis, NC 28081

^i^Institute for Advancing Health through Agriculture, Texas A&M University, College Station, TX 77845

^j^School of Exercise and Nutritional Sciences, San Diego State University, San Diego, CA 92182

^$^Corresponding Author:

Connie M. Weaver, Ph.D.

Distinguished Research Professor

School of Exercise and Nutritional Sciences

San Diego State University

5500 Campanile Drive

San Diego, CA 92182

765.412.2695| cmweaver@sdsu.edu

**Supplementary Figures:**

**Supplementary Figure 1.** Study design for OVX female rats. Treatments 1-4 equal 2.5%, 5%, 10% and 15% (w/w) freeze dried blueberry diet given in random order.


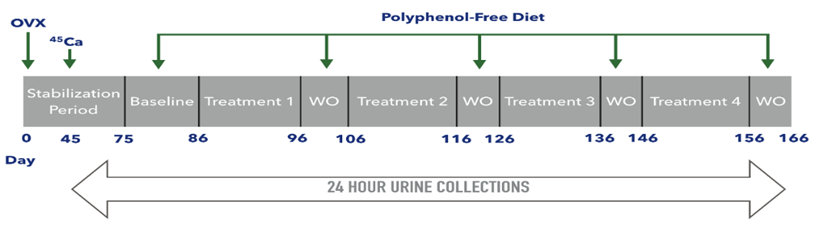


**Supplementary Figure 2.** Intakes of energy (A), macronutrients (B, C, D), soluble (E) and insoluble fiber (F), and bone-relevant micronutrients: calcium (G), vitamin D_2_ (H), magnesium (I), phosphorus (J), sodium (K), and potassium (L) in postmenopausal women at baseline (0 g/d) and after (A) or prior to the intervention (B) with low (17.5 g/d), medium (35 g/d), and high dose (70 g/d) of freeze-dried blueberry powder. Calcium and vitamin intakes include the amounts provided with the study supplement (Spectravite Advanced Formula; CVS/Pharmacy). Intakes of energy, carbohydrate, soluble, and insoluble fiber were significantly higher during the intervention with high dose of blueberry powder compared with baseline, whereas vitamin D intakes were lower during the treatment regardless of the blueberry powder dose. Data were analyzed by RM ANOVA followed by Dunnett’s post-hoc test. Data are means ± SD, **P* < 0.05, ***P* < 0.01, *n* = 14.


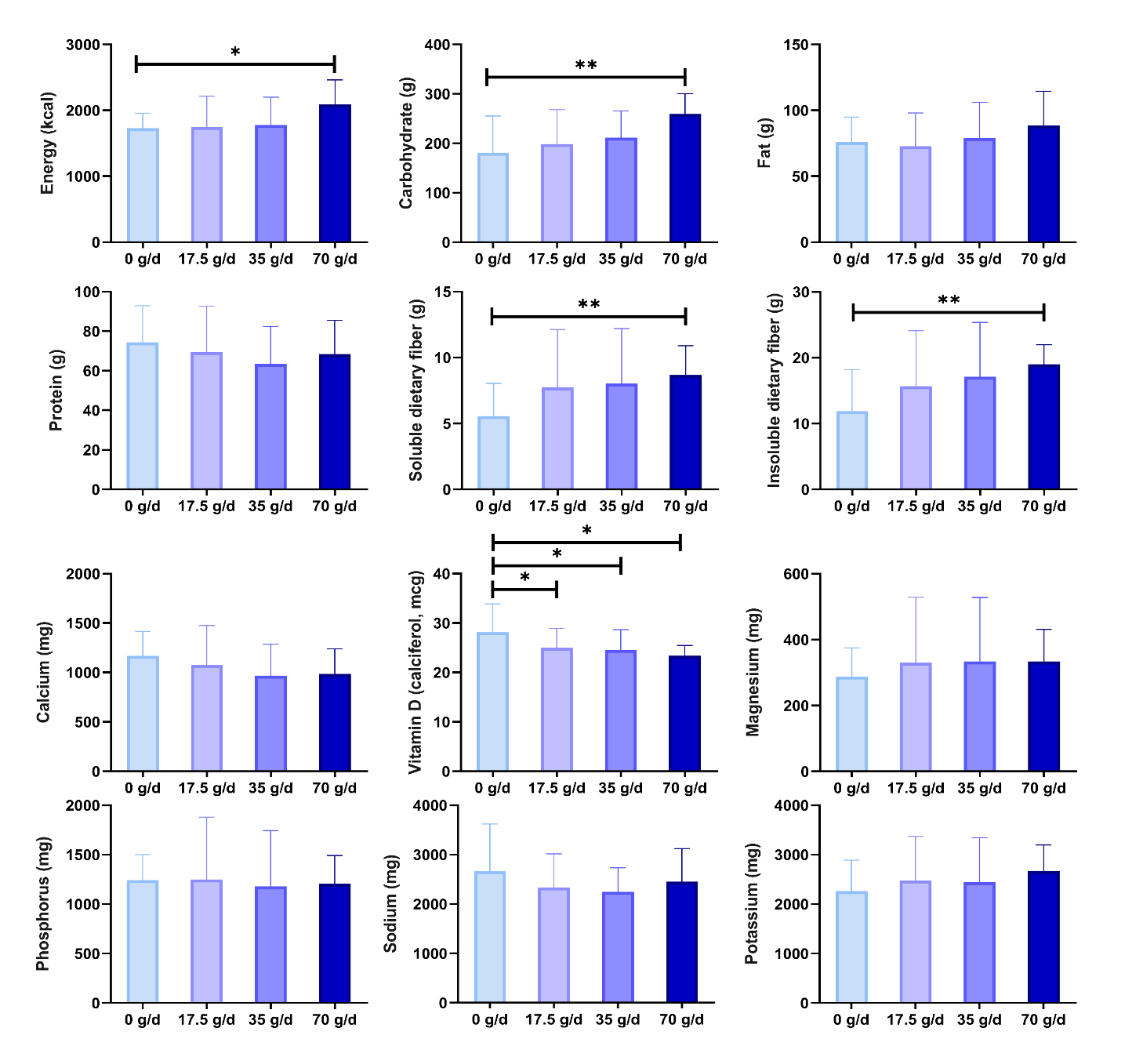


**A**

**B**

**C**

**D**

**E**

**F**

**G**

**H**

**I**

**J**

**K**

**L**
